# Activation of Human γδ T Cells by Cytosolic Interactions of BTN3A1 with Soluble Phosphoantigens and the Cytoskeletal Adaptor Periplakin

**DOI:** 10.4049/jimmunol.1401064

**Published:** 2015-01-30

**Authors:** David A. Rhodes, Hung-Chang Chen, Amanda J. Price, Anthony H. Keeble, Martin S. Davey, Leo C. James, Matthias Eberl, John Trowsdale

**Affiliations:** * Immunology Division, Department of Pathology, University of Cambridge, Cambridge Institute for Medical Research, Cambridge CB2 0XY, United Kingdom;; † Cardiff Institute of Infection & Immunity, School of Medicine, Cardiff University, Heath Park, Cardiff CF14 4XN, United Kingdom; and; ‡ Protein and Nucleic Acid Chemistry Division, Medical Research Council Laboratory of Molecular Biology, Cambridge CB2 0QH, United Kingdom

## Abstract

The three butyrophilin BTN3A molecules, BTN3A1, BTN3A2, and BTN3A3, are members of the B7/butyrophilin-like group of Ig superfamily receptors, which modulate the function of T cells. BTN3A1 controls activation of human Vγ9/Vδ2 T cells by direct or indirect presentation of self and nonself phosphoantigens (pAg). We show that the microbial metabolite (*E*)-4-hydroxy-3-methyl-but-2-enyl pyrophosphate binds to the intracellular B30.2 domain of BTN3A1 with an affinity of 1.1 μM, whereas the endogenous pAg isopentenyl pyrophosphate binds with an affinity of 627 μM. Coculture experiments using knockdown cell lines showed that in addition to BTN3A1, BTN3A2 and BTN3A3 transmit activation signals to human γδ T cells in response to (*E*)-4-hydroxy-3-methyl-but-2-enyl pyrophosphate and the aminobisphosphonate drug zoledronate that causes intracellular accumulation of isopentenyl pyrophosphate. The plakin family member periplakin, identified in yeast two-hybrid assays, interacted with a membrane-proximal di-leucine motif, located proximal to the B30.2 domain in the BTN3A1 cytoplasmic tail. Periplakin did not interact with BTN3A2 or BTN3A3, which do not contain the di-leucine motif. Re-expression into a BTN3A1 knockdown line of wild-type BTN3A1, but not of a variant lacking the periplakin binding motif, BTN3A1Δexon5, restored γδ T cell responses, demonstrating a functional role for periplakin interaction. These data, together with the widespread expression in epithelial cells, tumor tissues, and macrophages detected using BTN3A antiserum, are consistent with complex functions for BTN3A molecules in tissue immune surveillance and infection, linking the cell cytoskeleton to γδ T cell activation by indirectly presenting pAg to the Vγ9/Vδ2 TCR.

## Introduction

Major insights into the biology of γδ T cells, a population of unconventional or innate T cells characterized by their distinct TCR, have been made, linking their regulation to butyrophilin (BTN)-like molecules (1, 2). Work in mice has shown that a BTN-like molecule called Skint1 drives the selection and organ-specific homing of Vγ5/Vδ1 T cells associated with the murine skin (3). More recently, the activation of human Vγ9/Vδ2 T cells has been linked to BTN3A1. Vγ9/Vδ2 T cells respond specifically to phosphoantigens (pAg), the microbial isoprenoid precursor (*E*)-4-hydroxy-3-methyl-but-2-enyl pyrophosphate (HMB-PP), shared by many Gram-negative and Gram-positive bacteria as well as malaria parasites, and at much higher concentrations, to the related metabolite isopentenyl pyrophosphate (IPP), which is present in all living prokaryotic and eukaryotic cells (4, 5). Published reports show that BTN3A1 acts as an Ag presentation molecule for HMB-PP and IPP (6–8), but the mechanism remains poorly understood.

BTN3A1 has the structure of a type I receptor of the Ig superfamily and is part of a family of seven BTN receptors encoded by genes in the MHC (9). BTN molecules are composed of two Ig domains (IgV, IgC2), a single transmembrane domain, and a large carboxyl-terminal domain termed B30.2 (or PRYSPRY) located in the cell cytoplasm. There are three human *BTN3A* loci, *BTN3A1*, *BTN3A2*, and *BTN3A3*, and clear orthologs of BTN3A molecules, now called CD277, are absent from the mouse genome. Despite its similarity to B7 molecules, BTN3A1 was proposed to act not as a coreceptor or costimulatory molecule, but rather to directly present pAg to the γδ TCR in a manner analogous to MHC-restricted peptide presentation (8). However, this model of BTN3A1 function has been challenged by conflicting data, which show pAg binding to a positively charged pocket in the cytosolic B30.2 domain, and that BTN3A1 does not directly engage the γδ TCR (7, 10). This contradictory picture has emerged as a result of the complexity of the system and in particular by the use of endogenous and exogenous routes of Ag delivery in in vitro assays. Although the identification of BTN3A1 as an essential component in the control of human Vγ9/Vδ2 T cells has provided an important insight into the regulation of this T cell subset, clarification of where pAg binds, the role of the three BTN3A isoforms, and identification of BTN3A1 interacting molecules is required to resolve the molecular basis of the response.

## Materials and Methods

### Expression constructs

Primer sequences are listed in Supplemental Table I. BTN expression constructs were produced using pFLAG vector. For yeast two-hybrid bait and GST fusions, BTN3A cytosolic tails were cloned into pGBKT7 and subcloned into pGEX4T1. Periplakin (PPL) construct PPL B7 was prepared by subcloning the pGADT7 insert into pcDNA3hisC. PPL1-495 hemagglutinin (HA) was from L. Sevilla (Cancer Research UK Cambridge Institute, Cambridge, U.K.). PPLBamH1 was prepared by subcloning the 1.1-kb BamH1 fragment from clone B7 into pcDNA3hisB. DNA encoding specific short hairpin RNA (shRNA) directed to BTN3A and periplakin were cloned into pHR-SIREN/puro (a gift from Dr. Nick Matheson, Cambridge Institute for Medical Research, Cambridge, U.K.) using BamHI/EcoRI. Virus particles were produced by cotransfection with gag-pol pCMV8.91 and VSV-G env pMDG plasmids into 293T cells. Supernatant harvested after 48 h was filtered and used to transduce HeLa cells. Clones were selected by puromycin (1 μg/ml). Sequences targeted by shRNA were: shBTN3A: 5′-CGTGTATGCAGATGGAAAG-3′; shBTN3A1: 5′-CGTTGATGTGAAGGGTTAC-3′; shBTN3A2: 5′-CGTCGAAGTGAAGGGTTAT-3′; shBTN3A3: 5′-GCTGCAACAGAGCAAGAAA-3′; shPPL#1: 5′-ACCAGAAGAACCTGCTAGA-3′; shPPL#2: 5′-GTGGAAGTCAAAGAGGTGA-3′; shPPL#3: 5′-GACTGATCGAAAGGTGTGA-3′. BTN3A1 cDNA was cloned into the pHRsinIRES.GFP lentiviral vector using BamH1/Not1. Internal BamH1 sites were mutated neutrally to facilitate cloning (Quick Change, Stratagene). The site targeted by shBTN3A1 was also mutated. Site-directed mutagenesis was then used to create a series of variants targeting the B30.2 domain (H351R, W391A), periplakin interaction motif (Δexon5, ΔLL), and the asparagine-linked glycan (N115D). A stop codon was introduced in exon 8 to produce a ΔB30.2 truncation variant. For re-expression, HeLa shBTN3A1 cells were cotransduced with lentivirus-carrying variant BTN3A1 sequences. GFP^+^ cells were sorted using BD FACSAria cell sorter.

### Protein expression, purification, and isothermal titration calorimetry

BTN3A1 residues 310–513 and periplakin residues 133–530 (PPLBamH1) were expressed with an N-terminal His tag in C41 (DE3) *Escherichia coli* cells at 18°C overnight and purified by capture on Ni-NTA resin (Qiagen) followed by gel filtration in buffer containing 20 mM TRIS pH 8.0, 150 mM NaCl, 1 mM DTT. Fractions containing pure protein were pooled, concentrated to 20 mg/ml, and flash-frozen in liquid nitrogen. Isothermal titration calorimetry (ITC) experiments were conducted on a MicroCal ITC200. BTN3A1 protein was dialyzed overnight against buffer containing 50 mM TRIS pH 8.0, 150 mM NaCl, and 1 mM DTT. Periplakin protein and the ligands HMB-PP and IPP (Echelon Bioscience) were dissolved in this same dialysis buffer. To obtain optimal binding isotherms, we carried out ITC experiments using the indicated concentrations of BTN3A1 and ligands.

### Crystallization, data collection, structure determination, and refinement

Crystals were grown in 15% PEG 4K, 0.2M MgCl_2_, Tris pH 8.5. X-ray data were collected at 100 K on an in-house rotating anode X-ray generator. Data were processed using MOSFLM and the CCP4 suite. Structure was determined by molecular replacement using Phaser with 2IWG as a model (11). Model building was performed using Coot, and refinement was carried out using REFMAC5. Crystal structure data collection and refinement statistics are given (Supplemental Table II). B30.2 structure allocated accession code 4v1p in Protein Data Bank (http://www.rcsb.org/pdb/home/home.do).

### Ab production

GST fusion proteins were produced in *E. coli* strain BL21(DE3) grown in 2×TY medium at 22°C and purified using glutathione Sepharose (Amersham). For immunization, bound protein was eluted from washed beads using reduced glutathione (10 mg/ml in 50 mM Tris-base pH 10.2). Purified protein (1 mg/ml in 1×PBS) was used to immunize rabbits (Covalab). Immune sera (5 ml/45ml 1×PBS) were negatively selected twice over glutathione Sepharose columns preloaded with GST fusion of the reciprocal BTN3A B30.2 protein, then immunoaffinity purified. Antisera were used in immunohistochemistry and immunofluorescence at 10 μg/ml and immunoblotting at 1 μg/ml.

### Immunohistochemistry

Immunohistochemistry in paraffin-embedded tissue microarrays was performed by G. Flack and A. Warford, at the Atlas of Protein Expression Group, Wellcome Trust Sanger Institute, Wellcome Trust Genome Campus, Hinxton, Cambridgeshire, U.K., as described previously (12). To establish whether tissue staining represented true Ab binding, we probed serial tissue sections with purified rabbit polyclonal preimmune serum. Nonspecific binding of detection reagents was also routinely assessed by processing tissue sections without primary Ab.

### Yeast two-hybrid screen

The Matchmaker GAL4 system (Clontech) was used according to manufacturer instructions. All yeast dropout selection media were prepared in-house (G. Chalkin, Media Kitchen, Cambridge Institute for Medical Research). The bait vector was cotransfected into yeast strain AH109 with a premade cDNA library of 2 × 10^6^ clones in pGADT7. Selection for two-hybrid interactions was by growth on quadruple dropout media with color selection (XαGal). Plasmid DNA from positive interactors were rescued and inserts were sequenced.

### Immunoblot and pull-down assays

Cell lysates were prepared in buffer (50 mM Tris-Cl pH 7.5, 150 mM NaCl, 1% Triton-X, 2 mM PMSF, 5 mM iodoacetamide, EDTA-free protease inhibitor) by incubation for 10 min at 4°C, then precleared by centrifugation. For immunoblots (IBs), proteins were solubilized in SDS-PAGE buffer (5 min, 95°C) and separated in 10% SDS-PAGE gels, transferred to Immobilon-P membrane, blocked (5% Marvel/PBS 0.1% Tween 20), and incubated for 1 h with primary and HRP-conjugated secondary Abs. Blots were visualized with ECL reagent. Monoclonal M2 anti-FLAG Ab (Sigma Aldrich) was used. For pull-down assays, cell lysates were incubated at 4°C with mixing for 2 h with glutathione-Sepharose beads loaded with GST B30.2 domain fusion proteins. After washing, eluted proteins were analyzed by IBs and probed with appropriate Ab. Cell fractionation was carried out using the Qproteome cell compartments kit (Qiagen). Periplakin antiserum TD2 was from L. Sevilla (Cancer Research UK Cambridge Institute).

### Tissue culture

MCF-7, A431, EJ28 (kind gifts from L. Sevilla), HeLa, 293T, and cos-7 cell lines were maintained in RPMI 1640 medium plus 10% FCS, penicillin/streptomycin (100 U/ml), and l-glutamine (2 mM). Cells growing in six-well plates were transfected with DNA expression constructs using Fugene. For RT-PCR, Superscript III (Invitrogen) was used to produce first-strand cDNA from total RNA (200 ng) prepared from cultured cells using RNeasy (Qiagen). Amplification was carried out using Biomix Taq polymerase (Bioline). RT-PCR products were analyzed by gel electrophoresis and cloned using Zero-Blunt Topo (Invitrogen).

### T cell assays

Vγ9/Vδ2 T cells were expanded from healthy donor PBMCs with 1 μM zoledronate (Zometa; Novartis) and 100 U/ml IL-2 (Proleukin; Chiron) for 14 d. At the end of the culture period, γδ T cells were further enriched by negative selection with a modified human γδ T cell isolation kit that removes B cells, αβ T cells, NK cells, dendritic cells, stem cells, granulocytes, and monocytes (Stem Cell Technologies). All γδ T cells used in this study were >90% CD3^+^ Vγ9^+^ as determined by FACS analysis. Target HeLa cells were pulsed overnight with 10 μM zoledronate, washed extensively, and cocultured with γδ T cells at a ratio of 1:1 (2 × 10^4^ cells each). Alternatively, untreated HeLa cells were cocultured with γδ T cells in the absence or presence of 10 nM HMB-PP (a gift from H. Jomaa, Giessen, Germany). The amount of IFN-γ secreted into the culture supernatant over 24 h was measured by ELISA (eBioscience). Mobilization of CD107a onto the cell surface over the first 5 h of coculture was determined using a PE-conjugated anti-CD107a Ab (H4A3; BD Biosciences) in the presence of monensin at a 1:200 dilution (GolgiStop). Cells were acquired on a FACS Canto II and analyzed with FlowJo.

## Results

### pAg bind to the BTN3A1 B30.2 domain

**FIGURE 1.**
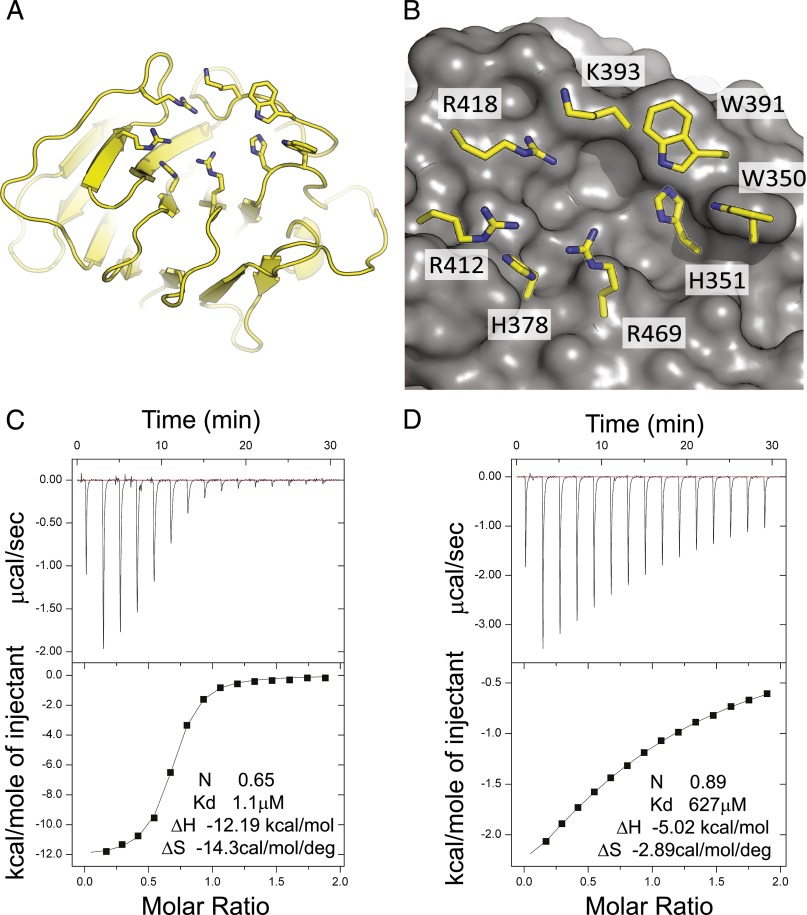
Structure of the BTN3A1 B30.2 domain. (**A**) Overview of the fold is shown with binding-site residues shown in stick representation. (**B**) A close-up view of the proposed pAg binding site showing the molecular surface and important interface residues (10). (**C** and **D**) ITC was used to show binding of pAg to BTN3A1 B30.2 domain. HMB-PP→BTN3A1 at 0.639 mM into HMB-PP at 0.064 mM; IPP→IPP at 6 mM into BTN3A1 at 0.597 mM.

The possibility that pAg interact with the B30.2 domain of BTN3A1 was first proposed by Harly et al. (7). As a result of our interest in the B30.2 domain of TRIM21, a high-affinity cytosolic FcR (11), we favored this hypothesis. The B30.2 domain represents a credible structure for binding small molecules in the cell cytoplasm with high affinity. Therefore, we investigated the structure and binding interactions of the BTN3A1 B30.2 domain (Fig. 1A, 1B). The crystal structure, like that of the B30.2 domains of TRIM21 and TRIM5 (13), shows it to be composed of layered antiparallel β sheets with variable loops, a structure homologous to the Ig fold. The juxtaposition of B30.2 domain variable loop residues forms the specific binding interface. The BTN3A1 B30.2 domain structure has also been presented by Sandstrom et al. (10), who identified a credible pocket implicated in binding pAg ligand, characterized by a cluster of positively charged residues (Fig. 1B). In addition to the residues highlighted by Sandstrom et al. (10), particularly arginine residues at R412, R418, and R469, histidines H351 and H378 and the single lysine K393, our apo structure suggests a role in coordinating Ag for tryptophan residues W350 and W391. In particular, the indole of W391 may form a hydrogen bond with phosphates in the bound ligand (Fig. 1B).

Binding of HMB-PP and IPP to the BTN3A1 B30.2 domain was investigated by ITC. HMB-PP was found to bind with an affinity of 1.1 μM (Fig. 1C) and IPP with an affinity of 672 μM (Fig. 1D), values that are similar to those reported (10) and that help explain the 1000- to 10,000-fold difference in bioactivity of the two molecules on human γδ T cells (5). Therefore, our results extend the findings of Sandstrom et al. (10) and confirm the intracellular interactions of pAg to the B30.2 domain.

### Identification of periplakin as a BTN3A1 interacting molecule

Next, we set out to explore the significance of the BTN3A1 cytoplasmic tail, including the B30.2 domain, by identifying interacting molecules using yeast two-hybrid experiments. The BTN3A1 cytoplasmic tail was cloned into the pGBKT7 bait vector and used to screen a human cDNA library. Twelve clones showed consistent yeast two-hybrid interactions. Further validation on dropout media with color selection against empty bait vector pGBKT7 identified six BTN3A1-specific two-hybrid interacting clones, which were sequenced.

Candidate clones were validated using GST pull-down assays. Inserts in the pGADT7 library vector were subcloned into pcDNA3 to give anti-Xpress fusion constructs, which were transfected into Cos-7 cells. Cell lysates were incubated with glutathione Sepharose beads preloaded with B30.2 domain GST fusion proteins. After washing, bound protein was eluted and analyzed by IB using anti-Xpress. Clone B7 showed positive interactions by these methods, representing aa 126–657 of periplakin, a 195-kDa cytosolic protein of the cytoskeleton-associated plakin family (14, 15). Interaction was specific to BTN3A1 because periplakin did not interact with BTN2A1 or BTN3A3 fusion protein in pull-down assays (Supplemental Fig. 1A).

**FIGURE 2.**
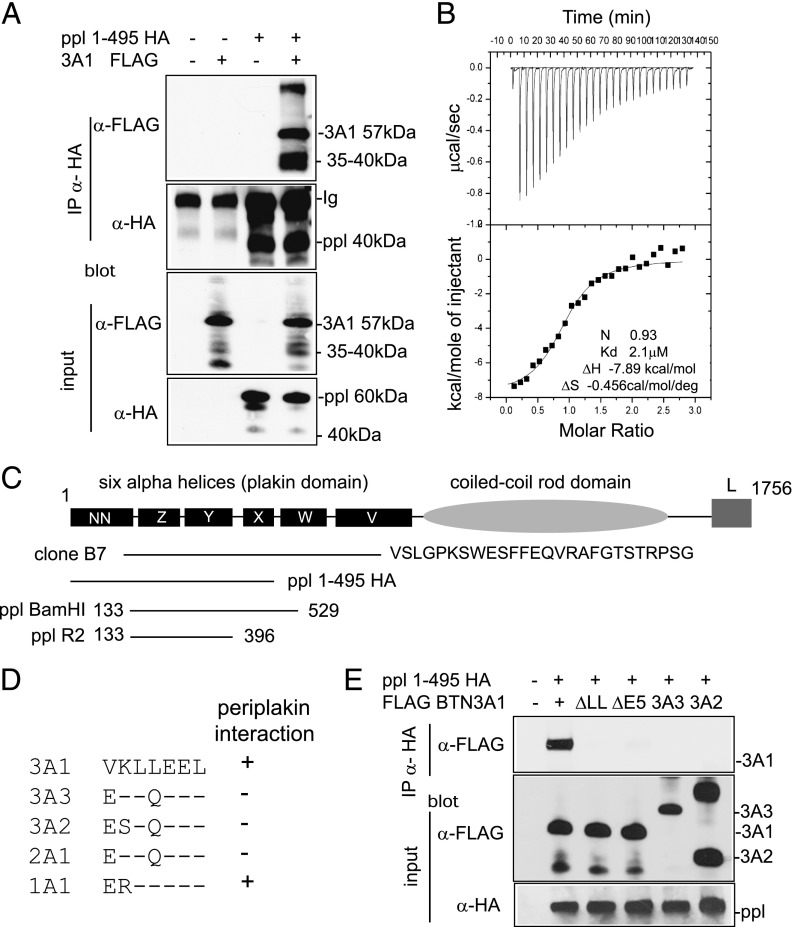
Interaction of BTN3A1 with periplakin. (**A**) Interaction of periplakin and BTN3A1 in vivo by IP. 293T cells were transfected with expression constructs PPL1-495 HA and FLAG BTN3A1 either alone or together. Protein complexes were recovered by IP using anti-HA Ab and analyzed by IB using anti-FLAG (*upper panel*) or anti-HA (*second panel*) Abs. FLAG BTN3A1 was recovered from anti-HA IP only in the presence of PPL1-495 HA. *Lower panels* show input lysates analyzed by IB. Protein bands with molecular weights lower than the introduced expression constructs were detected for both BTN3A1 and periplakin in IP experiments, as shown, which likely represents postlysis proteolytic cleavage of proteins. (**B**) ITC was used to confirm thermodynamically favorable interaction between purified BTN3A1 and PPL BamH1. Binding affinity in the low-micromolar range (2.1 μM) was calculated. (**C**) Diagram of domain structure of periplakin (14) showing expression constructs used. Clone B7 represented periplakin aa 126–657 plus 26 additional amino acids, showing similarity to XP_001098863 periplakin-like sequence from *Macaca mulatta*. (**D**) Alignment of amino acids (306–312) encoded by exon 5 of BTN3A1 compared with other BTN proteins. (**E**) Periplakin interaction via the di-leucine motif in BTN3A1. IP of wild-type BTN3A1 and variants lacking either exon 5 or the di-leucine motif. 293T cells were transfected with the indicated BTN3A expression constructs together with PPL1-495 HA. Periplakin and associated proteins were recovered from cell lysates by IP using anti-HA Ab and analyzed by IB using anti-FLAG. *Bottom panels* show IB of input lysates using anti-FLAG and anti-HA acting as a loading control.

Immunoprecipitation (IP) was used to confirm the periplakin–BTN3A1 interaction in vivo (Fig. 2A). Expression constructs FLAG BTN3A1 and PPL1-495 with HA tag were transfected individually or together into 293T cells. Anti-HA Ab was used to recover protein complexes, followed by IB using FLAG Ab. FLAG BTN3A1 was recovered from cell lysates by IP using HA Ab, only in the presence of PPL1-495 HA (Fig. 2A, *upper panel*). The two *lower panels* of Fig. 2A show IB of cell lysates to confirm protein input.

ITC experiments were used to investigate the interaction of BTN3A1 and periplakin proteins in vitro. A binding affinity of 2 μM was calculated for the BTN3A1 cytoplasmic tail interacting with a periplakin fragment representing the XYZ α helices of the plakin domain, with 1:1 stoichiometry (Fig. 2B). Using a series of BTN3A1 deletions produced by site-directed mutagenesis, the periplakin interaction site was localized to aa 305–310, overlapping with exon 5 of the BTN3A1 cDNA sequence (Supplemental Fig. 1B).

An interaction of periplakin with a BTN3A1 splice variant, 3A1v2, which lacks the B30.2 domain (NM_194441), was demonstrated by GST pull-down assay and IP (Supplemental Fig. 1C, 1D). Using ITC, a binding affinity of 0.58 μM was calculated for the 3A1v2 cytoplasmic tail interaction with periplakin (Supplemental Fig. 1E), a slightly increased affinity than that found for the full-length BTN3A1/periplakin interaction. The results showed that periplakin interacted with full-length BTN3A1 (Fig. 2) and a truncated splice variant BTN3A1v2 (Supplemental Fig. 1), indicating that the B30.2 domain was not required for binding. Periplakin was also shown to interact with BTN1A1, the BTN molecule expressed in human milk (Supplemental Fig. 1D).

Periplakin is a large cytosolic protein of 1756 aa, composed of six α helices (the plakin domain) and a large coiled-coil rod domain (Fig. 2C). Clone B7, identified by yeast two-hybrid assays, represented a splice variant lacking the rod domain but covering the plakin domain. The minimal binding interfaces were identified between aa 396–495, containing the central X region of the plakin domain of periplakin interacting with exon 5 of BTN3A1.

A comparison of the 7 aa encoded by exon 5 from BTN molecules showed a di-leucine motif in BTN1A1 and BTN3A1, the only molecules to bind periplakin, not found in BTN2A1, BTN3A2, or BTN3A3 (Fig. 2D). The interaction of periplakin with full-length BTN3A1 was compared with constructs carrying deletions of either the di-leucine or exon 5 (Fig. 2E). Constructs for BTN3A2 and BTN3A3 were also included. Periplakin interacted with full-length BTN3A1 alone and not with any of the other constructs. Therefore, periplakin interacts with the di-leucine motif in the juxtamembrane domain of BTN3A1, proximal to the B30.2 domain, a sequence motif also found in BTN1A1.

### Expression of BTN3A in human cells and tissues

**FIGURE 3.**
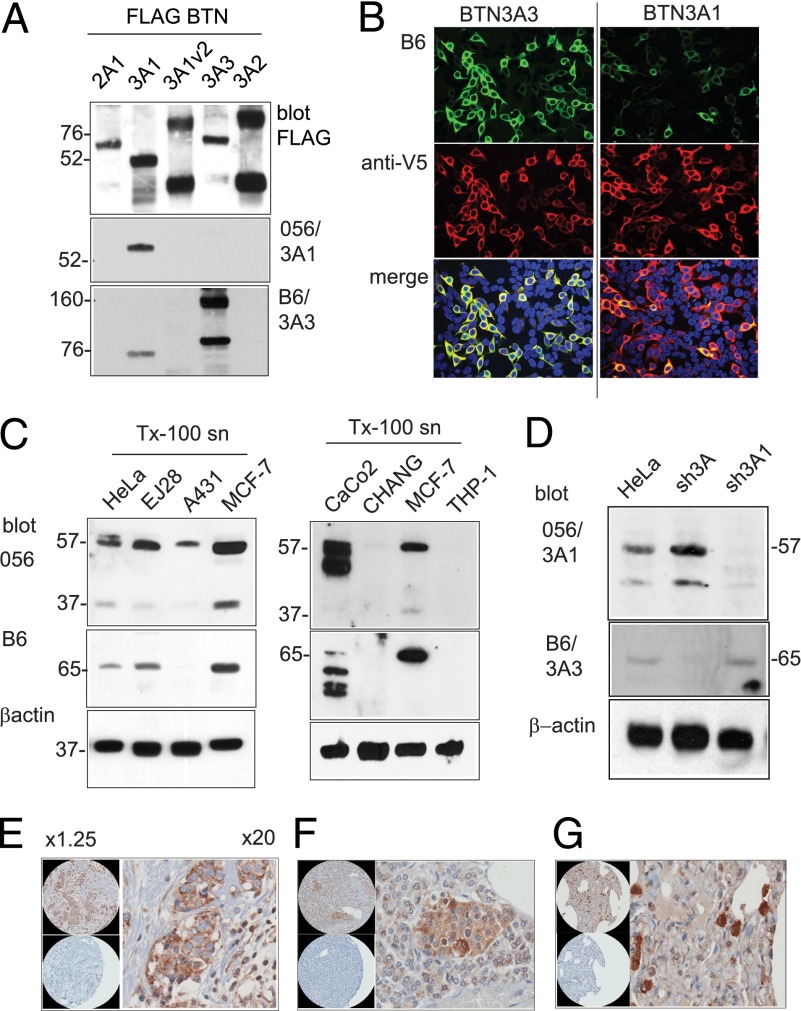
BTN3A protein expression in human cells and tissues. (**A**) 293T cells were transfected with pFLAG BTN expression constructs. Cell lysates were analyzed by nonreducing SDS-PAGE and IB using anti-FLAG and rabbit anti-B30.2 domain antisera 056 and B6 and goat anti-rabbit HRP secondary Abs. BTN3A1v2 is a truncated molecule lacking the B30.2 domain. Protein molecular mass in kDa. (**B**) Immunofluorescence microscopy of 293T cells transfected with expression constructs for BTN3A3 and BTN3A1. Fixed and Triton X-100 permeabilized cells (0.1%) were stained with antiserum B6/goat anti-rabbit Alexa 488 Abs (green) and carboxyl-terminal V5/goat anti-mouse Alexa 568 (red). Original magnification ×20. (**C**) BTN3A protein expression by IB of Triton X-100 detergent cell lysates from human epithelial carcinomas cell lines, as shown, probed with antisera 056 and B6 and goat anti-rabbit HRP secondary Abs. Loading control by β-actin Ab. (**D**) BTN3A expression in HeLa cells selected for expression of BTN3A shRNA knockdown vectors. Cell lysates were analyzed by IB with antisera 056 and B6 and by β-actin Ab. (**E**–**G**) Immunohistochemistry on human tissue array probed with Ab B6. Original magnification ×20. (E) Breast tumor tissue. (F) Islets of Langerhans. (G) Macrophages in the lung. Control panels [*lower panels* in (E)–(G)] were serial tissue sections stained with preimmune serum.

To assess protein expression from individual *BTN3A* loci, we produced Abs to B30.2 domains. BTN3A1 and BTN3A3 B30.2 domains as GST fusion proteins were used for immunization. This approach could not be used for BTN3A2, which does not have a B30.2 domain. IB of cell lysates from 293T cells transfected with FLAG-tagged BTN3A constructs was used to assess antisera specificity (Fig. 3A). Anti-FLAG Ab IB, used as a loading control, showed that FLAG BTN3A proteins in some cases formed dimeric complexes at high expression levels. Antiserum 056 was confirmed to be specific for BTN3A1, whereas antiserum B6 was predominantly against BTN3A3, with cross-reactivity with BTN3A1. B6 antiserum showed similar staining specificity in immunofluorescence microscopy of transfected 293T cells, recognizing predominantly BTN3A3, with cross-reactivity to BTN3A1 (Fig. 3B).

To determine BTN3A protein expression, we prepared and analyzed detergent lysates from human tumor-derived cell lines by SDS-PAGE followed by IB using the B6 and 056 antisera. BTN3A1 and BTN3A3 were expressed at low abundance in most lines tested at the expected molecular masses of 57 and 65 kDa, respectively, with the two proteins showing identical distribution (Fig. 3C). A lower molecular mass band of 37 kDa was evident using the BTN3A1-specific Ab 056, particularly in human breast adenocarcinoma MCF-7 cells, indicative of alternative splicing or proteolytic cleavage of BTN3A1.

HeLa cell lines expressing shRNA vectors targeting BTN3A transcripts were produced and cell lysates analyzed by IB, to confirm specificity of antisera (Fig. 3D). The sh3A1 vector was specific for BTN3A1, because antiserum 056 cross-reacting bands were depleted in this cell line. The sh3A vector efficiently knocked down expression of BTN3A3, recognized by B6 antiserum, but did not affect expression of BTN3A1.

The B6 antiserum gave specific results by IB and immunofluorescence microscopy, indicating that it would be useful in assessing protein expression in tissues. The B6 antiserum was used in immunohistochemistry against paraffin-embedded human tissue sections. Widespread epithelial and tissue macrophage cell staining was detected (Fig. 3E–G and Supplemental Fig. 2). Tumor sections showed more intense staining than that observed in normal epithelium, for example, in breast tumor tissue sections (Fig. 3E). There was notable staining of endocrine tissues including pancreatic islets (Fig. 3F). Macrophages in the spleen, liver Kupffer cells (Supplemental Fig. 2), and lung macrophages (Fig. 3G) also showed strong staining.

### Activation of γδ T cells requires multiple BTN3A isoforms

**FIGURE 4.**
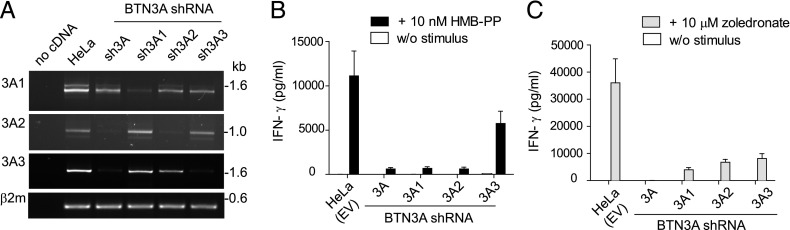
BTN3A-dependent activation of γδ T cells. (**A**) BTN3A knockdown by expression of shRNA targeting each isoform. Full-length BTN3A transcripts were amplified by RT-PCR from cDNA derived from HeLa BTN3A knockdown cell lines. Amplification of β_2_microglobulin was used as a template control. RT-PCR products were analyzed by gel electrophoresis. Size in kb. (**B** and **C**) Coculture of γδ T cells with BTN3A knockdown lines and empty vector control (HeLa EV). T cell activation was induced by (B) 10 nM HMB-PP in coculture or (C) pretreatment of HeLa cells with 10 μM zoledronate. IFN-γ secretion into culture medium was detected by ELISA. Data shown are mean values obtained from four independent experiments using γδ T cells from PBMCs of three individual donors, with error bars (SEM). w/o, without pAg.

BTN3A1 was previously shown to transmit activation signals to Vγ9/Vδ2 T cells in the presence of pAg stimulation (16). To test the role played by BTN3A1 in activating Vγ9/Vδ2 T cells, we used the two HeLa cell lines expressing shRNA vectors targeting BTN3A molecules characterized in Fig. 3D. Specific knockdown by these shRNAs was confirmed by RT-PCR (Fig. 4A). In coculture experiments, sh3A and sh3A1, together with shRNA empty vector control (HeLa EV), were pulsed with zoledronate to induce intracellular accumulation of IPP and incubated overnight with expanded γδ T cells. To test the effect of exogenously provided pAg, we also cocultured HeLa cells and γδ T cells in the absence or presence of the microbial metabolite HMB-PP. Culture supernatants were analyzed by ELISA for IFN-γ (Fig. 4B, 4C). These experiments confirmed the critical role played by BTN3A1 in γδ T cell activation, but showed additional complexity dependent upon the T cell activation marker and the stimulus used. In addition, although our analysis of protein expression showed that the sh3A line expressed BTN3A1, but not BTN3A3, whereas the sh3A1 line expressed BTN3A3, but not BTN3A1 (Fig. 3D), the data were also consistent with a requirement for multiple BTN3A isoforms in γδ T cell activation.

Because of the apparent discrepancy of these findings with published reports that excluded a role for BTN3A2 and BTN3A3 in γδ T cell regulation (7), two further knockdown lines, sh3A2 and sh3A3, which target specifically BTN3A2 and BTN3A3, respectively, were produced. Because anti-BTN3A Ab CD277 stained HeLa cells poorly, we do not have Abs specific to BTN3A2 and we could not consistently show presence/absence of protein using BTN3A antisera; knockdown lines were analyzed by RT-PCR. cDNA amplification to detect full-length transcripts for each of the BTN3A isoforms showed that shRNA vectors were specific to their targeted transcript (Fig. 4A). When tested in γδ T cell assays, the production of IFN-γ, induced by either soluble HMB-PP (Fig. 4B) or in response to zoledronate-pulsed target cells (Fig. 4C), was efficiently blocked in all BTN3A knockdown lines compared with the vector control line, with only the BTN3A3 line showing a reduced knockdown phenotype in response to HMB-PP. Taken together, the results demonstrated a role for all three BTN3A isoforms in regulating pAg-induced γδ T cell activation.

### Role of periplakin in γδ T cell activation

**FIGURE 5.**
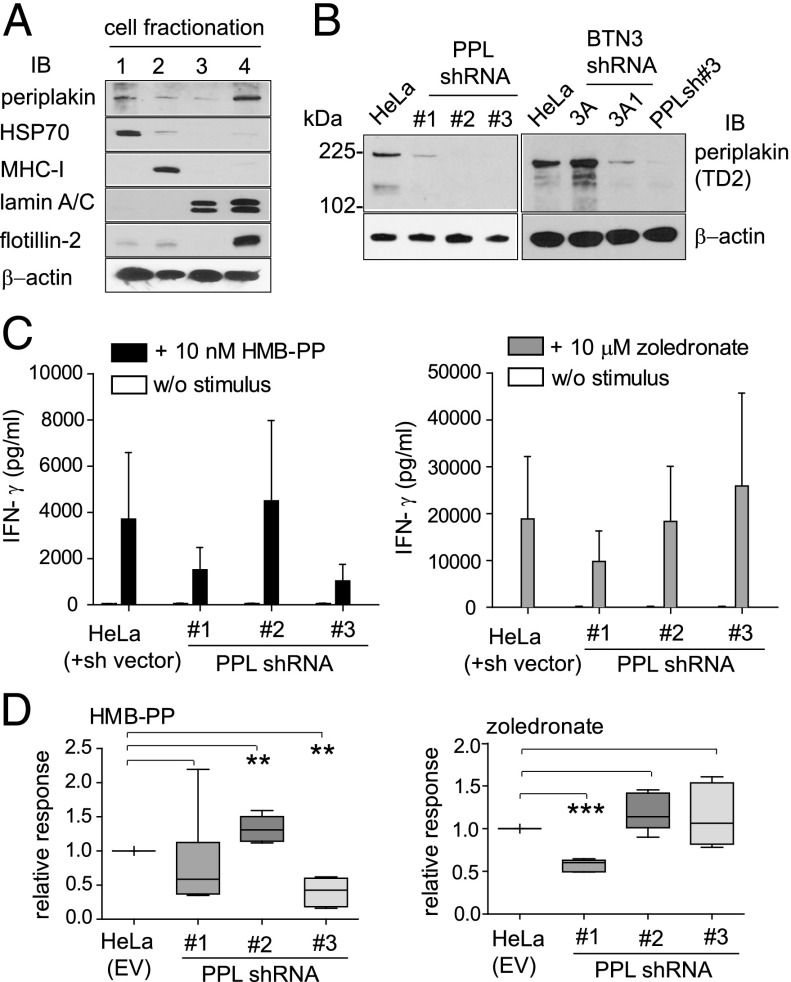
Role of periplakin in γδ T cell activation. (**A**) Cell fractionation based on detergent solubilization. Fractions from HeLa cells (2 × 10^6^) were analyzed by SDS-PAGE and IB using antiperiplakin antiserum TD2. Control Abs were used to show efficiency of fractionation into (*1*) cytosolic, (*2*) membrane, (*3*) nuclear, and (*4*) cytoskeletal/detergent-resistant membrane fractions. Anti–β-actin Ab acts as a loading control. (**B**) Periplakin knockdown by expression of shRNA in HeLa cells (*left panel*). Cytoskeletal/detergent-resistant membrane fractions were prepared and analyzed by IB using antiperiplakin antiserum. IB of detergent-resistant fractions in BTN3A and periplakin knockdown cells (*right panel*). Anti–β-actin Ab acts a loading control in each case. (**C**) Coculture of γδ T cells with periplakin shRNA knockdown lines PPLsh#1, PPLsh#2, and PPLsh#3. T cell activation was induced using 10 nM HMB-PP (*left panel*) or 10 μM zoledronate (*right panel*), and IFN-γ secretion into culture medium was detected by ELISA. HeLa EV empty vector control. Data shown are representative of five independent experiments, with error bars (SEM). (**D**) Analysis of data from (C) of γδ T cell stimulation induced from PPL knockdown cells. Box plots (depicting 25th percentile, median, 75th percentile, and highest/lowest data point whiskers) of relative change in IFN-γ responses compared with vector control (HeLa EV), from *n* = 5 determinations. Differences between groups were assessed by one-way ANOVA. A *p* value <0.05 was considered significant. **p < 0.01, ***p < 0.001. w/o, without pAg.

The interaction of BTN3A1 with periplakin (Fig. 2) implied a role for this cytoskeletal adaptor in the regulation of γδ T cell responses. Because of limitations in the choice of specific Abs and the fact that both proteins are of low abundance, a direct demonstration of the interaction of endogenous BTN3A1 with periplakin by coimmunoprecipitation has not been possible. In addition, periplakin is a large protein of 195 kDa associated with cytoskeletal structures generally resistant to detergent solubilization. To detect periplakin consistently, we used cell fractionation based on detergent solubilization to produce soluble, membrane, nuclear, and cytoskeletal/detergent resistance membrane protein fractions (Fig. 5A). Fractions from 2 × 10^6^ HeLa cells were analyzed by SDS-PAGE and probed with periplakin Ab. A series of control Abs was used to demonstrate the efficiency of fractionation using this protocol. Periplakin was found to be generally distributed in cell fractions, but detected most robustly in the cytoskeletal/detergent-resistant membrane fraction, which was also positive for flotillin-2, a marker for caveolae or lipid raft domains (Fig. 5A).

To address the role of the BTN3A1–periplakin interaction on a functional level, we produced HeLa cell lines expressing shRNA targeting different regions of periplakin (PPL). Cytoskeletal/detergent resistance membrane fractions were isolated and analyzed by IB using antiperiplakin antiserum. All three PPL shRNA lines showed reduced or absent levels of periplakin (Fig. 5B, *left panel*). When analyzed similarly, the sh3A1 knockdown line showed reduced levels of periplakin, indicating that expression of periplakin may be linked to BTN3A1, possibly by stabilization of the protein (Fig. 5B, *right panel*).

The effect of periplakin on the production of IFN-γ by activated γδ T cells was analyzed using the periplakin knockdown lines in coculture experiments (Fig. 5C). Compared with the vector control (HeLa EV), two knockdown lines, PPLsh#1 and PPLsh#3, showed reduced IFN-γ production in response to HMB-PP (Fig. 5C, *left panel*). The PPLsh#2 line, in contrast, showed an increase in IFN-γ production. In response to IPP accumulation induced by zoledronate pretreatment (Fig. 5C, *right panel*), PPLsh#1 reduced IFN-γ production, PPLsh#2 showed no change, and PPLsh#3 showed a slight increase in IFN-γ levels. The results, which were consistent over multiple experiments (*n* = 5; Fig. 5D), showed that periplakin knockdown did not consistently block, but caused dysregulated γδ T cell responses to pAg exposure, as assessed by IFN-γ production. There was variation in the response to the different pAg, exogenous HMB-PP and endogenous IPP induced by zoledronate, by individual PPL knockdown lines that appeared to be dependent on which part of the periplakin molecule was targeted by shRNA.

**FIGURE 6.**
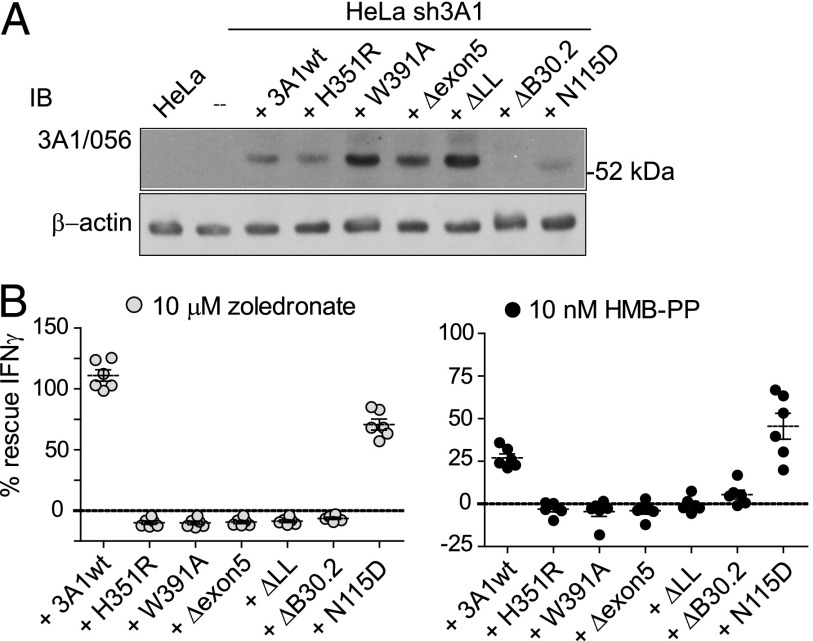
Activation of γδ T cells requires the B30.2 domain and periplakin interaction motif. (**A**) Analysis of re-expression lines using anti-BTN3A1 Ab 056. IB analysis of total protein (10 μg) from Triton-X100 detergent lysates from HeLa, HeLa sh3A1 knockdown line, and the parental sh3A1 cell line transduced additionally with IRES.GFP lentivirus–expressing wild-type BTN3A1, H351R, W391A, Δexon5, ΔLL, ΔB30.2, and N115D variants. Duplicate IB using β-actin acts as loading control. Endogenous BTN3A1 protein was not detectable by 056 antiserum in the untransfected HeLa cell lysate in these experimental conditions. BTN3A1 ΔB30.2 variant protein was also not detected using the 056 antiserum. (**B**) Analysis of re-expression lines in γδ T cell assays. Graph shows percent (%) IFN-γ recovery response from each of the re-expression lines compared with the vector control HeLa EV (positive) and sh3A1 knockdown (negative) lines. Data produced using γδ T cells from six individual donors.

To find clearer evidence of a role for periplakin, we used transcript re-expression into the sh3A1 cell line, which had been rendered unresponsive in T cell assays by shRNA-suppressing endogenous BTN3A1. Re-expression of BTN3A1 was achieved using the lentivirus system, where expression was monitored using IRES-driven GFP. A series of BTN3A1 variant lines were produced, targeting the proposed pAg binding motif in the B30.2 domain (H351R, W391A), the site of periplakin interaction (Δexon5, ΔLL), and the asparagine-linked glycan in the Ig IgV domain (N115D). A ΔB30.2 domain truncation variant, carrying a stop codon in exon 8, was used as an additional control. BTN3A1 protein expression in the reconstituted lines was confirmed by IB with anti-BTN3A1 Ab 056. Endogenous BTN3A1 was not detected in the untransfected HeLa line in these experimental conditions (Fig. 6A). IFN-γ levels induced by the reconstituted lines, in the presence of zoledronate pretreatment (Fig. 6B, *left panel*) or HMB-PP in coculture (Fig. 6B, *right panel*), were compared with those from empty vector control HeLa EV (positive) and sh3A1 (negative) parental lines in γδ T cell assays. IFN-γ production from T cells was rescued by coculture with wild-type BTN3A1 and N115D re-expression, but not by the B30.2 domain variants H351R and W391A, nor by the Δexon5 and ΔLL deletions. The ΔB30.2 deletion variant was also unable to rescue T cell responses. In addition, the IFN-γ recovery response to HMB-PP by reconstituted wild-type cells was less efficient than for zoledronate. These data therefore provide functional confirmation of the critical requirement for the B30.2 domain, of key residues within this domain implicated in pAg binding and for the BTN3A1 periplakin interaction motif residues, in pAg-dependent activation of γδ T cells, with the likelihood that additional unidentified factors are required (17).

## Discussion

BTN3A1 has been shown to regulate Vγ9/Vδ2 T cells, a subset of human T cells that elicit immune responses to microbial infection and with potent antitumor activity (7, 8, 18). Activation of Vγ9/Vδ2 T cells is achieved specifically by presentation of low-m.w. pAg such as HMB-PP and IPP. Our results address directly the complex mechanism of pAg sensing by BTN3A1, confirming that both HMB-PP and IPP bind to the cytosolic BTN3A1 B30.2 domain with affinities of 1.1 and 672 μM, respectively, in line with affinities reported previously (10). What is less clear is how such binding is transmitted to the γδ TCR. BTN3A1 conformational change, redistribution in the cell membrane as a result of pAg binding, and recruitment of other molecules required for TCR engagement are the current proposals (10).

We demonstrate a specific interaction of BTN3A1 with the cytoskeletal adaptor protein periplakin. Published results indicate a role for the plakin protein family, including periplakin, in tissue homeostasis and regulation of immune responses in epithelium (19). pAg-dependent responses could be restored to a BTN3A1 knockdown cell line by re-expression of wild-type BTN3A1, but not by variants lacking the periplakin interaction motif, confirming a critical role for this motif in transmitting activation signals to γδ T cells. Assays in the presence of periplakin knockdown cells were less conclusive. We detected a block in T cell activation induced by HMB-PP in only two of the three periplakin knockdown lines, and responses to zoledronate treatment were also not blocked completely, with IFN-γ levels in some experiments being increased. Although the binding and re-expression data implicated periplakin as a component of the pathway controlling γδ T cell activation, the dysregulated responses showed that periplakin has more of a regulatory role and that other factors may compensate for its absence by binding the same motif on BTN3A1. Analysis of the BTN3A1 knockdown cell line using anti-periplakin Ab showed that periplakin protein was reduced to levels comparable with that observed using specific periplakin knockdown reagents (Fig. 5B). Although we could not confirm any effect of periplakin knockdown on BTN3A1 protein using anti-BTN3A antisera by the reciprocal experiment, the results indicated that periplakin expression may be dependent on coordinated expression of BTN3A molecules, adding further complexity to the mechanism. The genomic organization at the periplakin locus on human chromosome 16p13 could also predispose the molecule to alternative splicing (20), which may contribute to evasion of shRNA-mediated suppression, variation in expression according to cell type, or in recruitment of known periplakin-interacting proteins envoplakin, involucrin, or kazrin (21, 22). Our working model is that binding of pAg to the BTN3A1 B30.2 domain recruits periplakin to increase avidity interactions or synapse formation by, for example, anchoring or redistribution in the membrane, receptor clustering, or recruitment of other molecules into a signaling complex. It will be important to establish whether binding affinity is altered in any way, and such studies will inform attempts at crystallization of the periplakin/BTN3A1 or the trimolecular complex with pAg.

Recent nuclear magnetic resonance analyses confirm a conformational change in the B30.2 domain upon pAg binding, extending to the BTN3A1 membrane-proximal region where periplakin binds (23). Fluorescence recovery after photobleaching experiments showed BTN3A1 redistribution and anchoring in pAg-pulsed cells (10). Given our results, it is also feasible that periplakin–BTN3A1 interaction impacts negatively on γδ T cell activation, regulates uptake of exogenous HMB-PP in an endocytic compartment, or that γδ T cell activation is conveyed by a BTN3A-dependent signaling mechanism.

The N-terminal plakin domain of periplakin, composed of six spectrin repeat α-helices and central SH3 domain (24), interacted with the cytoplasmic tail of BTN3A1 (and BTN1A1) via a membrane-proximal di-leucine motif. Di-leucine is a well-characterized motif involved in sorting and endocytosis in the secretory pathway, controlling protein localization and trafficking. Canonical di-leucine motifs have an upstream acidic residue, positioned at -4 (D/ExxxLL) or -3 (DxxLL/LI), where x is any amino acid, and bind either the AP2 clathrin adaptor or GGAs, respectively (25, 26). In contrast, the BTN3A1 sequence (TMKQ**E**QSTRVK**LL**EEL) has an acidic residue at position -7, suggesting a molecular mechanism with characteristics of E-cadherin–p120-catenin pathways (27). Masking of the endocytic di-leucine motif by p120 stabilizes E-cadherin expression at the cell surface (28). Consistent with our model, periplakin recruitment to the BTN3A1 di-leucine motif may be required for T cell activation by acting similarly, to enhance stability of the BTN3A1–pAg complex.

BTN3A knockdown lines, produced in HeLa cells by stable expression of shRNA vectors, were used in coculture experiments with γδ T cells and activation markers analyzed. The results confirmed the critical role played by BTN3A1 in activation of γδ T cells by pAg stimulation, and in addition showed that BTN3A2 and BTN3A3 are also required. The BTN3A isoform knockdowns showed responses, particularly with regard to IFN-γ production (Fig. 4), which were in most cases comparable with BTN3A1 knockdown in our assays. These data appear to be a discrepancy between other published reports, which have excluded a role for BTN3A2 and BTN3A3. The pAg binding pocket residues identified in the B30.2 domain of BTN3A1 (10), including the tryptophan residues at W350 and W391 (Fig. 1B), are conserved in BTN3A3, suggesting that BTN3A3 may bind pAg. This possibility has been excluded (10) and we have confirmed that the BTN3A1 H351R variant is nonfunctional in T cell assays. In addition, BTN3A2 does not contain a B30.2 domain, and neither BTN3A2 nor BTN3A3 bind periplakin. However, the BTN3A isoforms show >95% amino acid identity across Ig domains, so it may be that BTN3A2 and BTN3A3 act more as chaperones to modulate BTN3A1 trafficking or in protein complex formation, requiring coordinated expression.

Our data are consistent with the model of BTN3A1 regulation of γδ T cells, for which there is considerable evidence (7, 10, 18, 23), that pAg binding is presented indirectly to the γδ TCR, and are not consistent with the direct binding model presented by Vavassori et al. (8). We have confirmed that HMB-PP, and with lower-affinity IPP, bind to the BTN3A1 B30.2 domain. Our refined model proposes that transmission of pAg binding from the cytosol to the γδ TCR requires, in addition to BTN3A1, the isoforms BTN3A2 and BTN3A3, recruitment of periplakin, and possibly other members of the plakin family of cytolinker proteins, which serve to anchor or stabilize a BTN3A signaling complex in the cell membrane. In this scenario, the cytosolic sensing of metabolic intermediates of the endogenous mevalonate and pathogen-specific nonmevalonate pathways by BTN3A molecules may not require that pAg access the lumen of the endoplasmic reticulum, as for canonical MHC-I Ag presentation.

## Supplementary Material

Data Supplement
