# Supplementary material for: Activation of Human γδ T Cells by Cytosolic Interactions of BTN3A1 with Soluble Phosphoantigens and the Cytoskeletal Adaptor Periplakin
Source: J Immunol. 2015 Jan 30;194(5):2390–8. doi: 10.4049/jimmunol.1401064 (PMC4337483; doi:10.4049/jimmunol.1401064)
Supplement: Data Supplement [file JI_1401064.zip › JI_1401064_Supplemental_Material_1.pdf]

Supplementary Table I
Sequences of DNA oligonucleotide primers.

NAME	SEQUENCE	COMMENT
3A1.FOR<br>3A1.REV	ATGAAAATGGCAAGTTTCCTGG<br>AAGCTTCGCTGGACAAATACTCAGGGCC	RT-PCR and cloning
3A2.FOR<br>3A2.REV	GAATTCCATAG**ATG**AAAATGGCAAGTT<br>GATATCGGCTGACTTATTGGTATCG	
3A3.FOR<br>3A3.REV	GAATTCCATAG**ATG**AAAATGGCAAGTT<br>AAGCTTGTAAAGTGCTTCAGTGCGTGCC	
BTN3B F<br>BTN3B R	AGCAGCATCTGTGATCATGAGAGGC<br>AGGAAGTTTCAGGTTGGTTCTGGGC	
BTN2B F<br>BTN2B R	CCTGATGCAGACGGCCTCTTCATGG<br>CCATGAAGAGGCCGTCAGCATCAGC	
ACTIN F<br>ACTIN R	GTGTAACGCAACTAAGTCATAGTC<br>CATGGATGATGATATCGCCG	
BTN2A1.F	GAATTCCCAGTTTATTGTCGTGGGGCCC	pFLAG cloning
BTN3.F	GAATTCCCAGTTTTCTGTGCTTGGA	
1A1.F<br>1A1.R	GATATCATGGCAGTTTTCCCAAGCTCC<br>GATATCAGGTGCCCCTTGGCTGGG	For pEF6. Template cDNA from I.Mather
2A1.GB.F<br>2A1.GB.R	GAATTCAAACTCCAAAAGGAAAAAAAGATTCTG<br>GTCGACCTATAGGCTCTGGTGGGTCCCCAC	For 2 hybrid vector pGBKT7 EcoR1/BamH1 B30.2 domains
3A1.GB.F<br>3A1.GB.R2	GAATTCGAGGAAAAAAAGACTCAGTTCAG<br>GTCGACTCACGCTGGACAAATACTCAGGG	
3A1V2.GST.F<br>3A1V2.GST.R	GAATTCGAGGAAAAAAAGACTCAGTTCAG<br>CTCGAGTTTAACAAAGTGGAGCCTCATC	EcoR1 /Xho1 into pGEX4T1<br>BTF5alt C term
3A3.GST.F<br>3A3.GST.R	GAATTCAAGGAAAAAATTGCTCTG<br>GTCGACGTAAAGTGCTTCAGTG	R1/Sal into pGEX4T1<br>then pGBKT7
3A1V2.GST.R	CTCGAGTTTAACAAAGTGGAGCCTCATC	
3A2.GST.F<br>3A2.GST.R	GCCGAATTCAAGGAAATAACTGCTCTGTC<br>GTCGACGGCTGACTTATTGGTATCG	R1/Sal into pGEX4T1<br>then pGBKT7
PPL.R1<br>PPL.R2	GCCGGATCCGCGGCCCTTGGCCTGCTG<br>GGATCCCTCCCGGCGGTACTTGAG	For truncations BamH1 pcDNA3hisB
PPL.F2<br>PPL.R3<br>PPL.R4	GATATCCTCAAGTACCGCCGGGAG<br>GGATCCGGCATCTCCGGGATTCTC<br>GGATCCGGCCTGGATGAAGGCTTC	Ppl deletions for pEF6<br>With EcoRV and BamH1
		Mutagenesis
3A1.LL.F<br>3A1.LL.R<br>3A1.E5.F<br>3A1.E5.R	GAACAAAGCACAAGAGTGAAGGAGGAACTCAGATGGAGAAG<br>CTTCTCCATCTGAGTTCCTCCTTCACTCTTGTGCTTTGTTC<br>GAAGCAAGAACAAAGCACAAGAAGATGGAGAAGTATCCAGTA<br>TACTGGATACTTCTCCATCTTCTTGTGCTTTGTTCTTGCTTC	3a1 del LL and exon 5
3A1.TOSH3.F<br>3A1.TOSH3.R	CAGCACTGGGTTCTGATCTTCACATTGAAGTGAAGGGT TACAAGGATGGAG<br>CTCCATCCTTGTAACCCTTCACTTCAATGTGAAGATCAG AACCCAGTGCTG	Change residues targeted by BTN3sh3 knockdown vector in 3A1
3A1ASN.F<br>3A1ASN.R	GGCTGCTCTCCGAATACACGACGTCACAGCCTCTGACAG<br>CTGTCAGAGGCTGTGACGTCGTGTATTCGGAGAGCAGCC	Mutation of 3a1 Asn 115 site to Asp
3A1BAM.F<br>3A1BAM.R	CAAGCCTGCGGATGTGATTCTAGATCCAAAAACAGCAAACC<br>GGTTTGCTGTTTTTGGATCTAGAATCACATCCGCAGGCTTG	Mutation of 3A1 B30 domain BamH1 site (Bam2) to facilitate #291 cloning using BamH1/Not1

Supplementary Table 2

Crystal structure data collection and refinement statistics.

	BTN3
**Data collection**	
Space group	P 21212
Cell dimensions	
a, *b*, *c* (Å)	44.62, 124.66, 38.82
α, β, γ (°)	90, 90, 90
Resolution (Å)	62.38-2.03 (2.14-2.03)
R_{merge}	0.086 (0.331)
I / σI	14.3 (3.6)
Completeness (%)	95.8 (95.8)
Redundancy	3.2 (3.2)
**Refinement**	
Resolution (Å)	62.38-2.03 (2.14-2.03)
No. reflections	14322
R_{work} / R_{free}	0.165/0.222 (0.17/0.19)
No. atoms	
Protein	1531
Ligand/ion	0
Water	119
B-factors	
Protein	31.3
Ligand/ion	-
Water	33.4
R.m.s. deviations	
Bond lengths (Å)	0.009
Bond angles (°)	0.947

*Values in parentheses are for highest-resolution shell.

Supplementary Figure 1

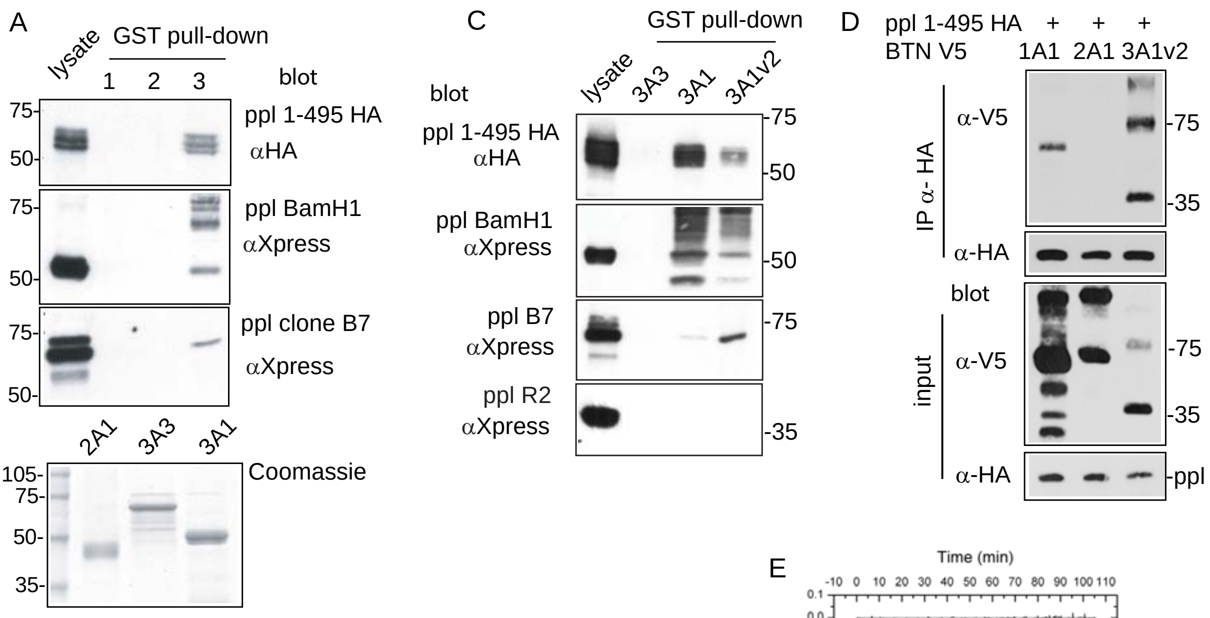

B

Construct	BTN3A1 seq.	Periplakin binding
BTN3EEK	275-513	0.8 µM
BTN3del1	280-513	0.8 µM
BTN3del2	285-513	2 µM
BTN3del3	290-513	2 µM
BTN3del4	295-513	2 µM
BTN3del5	300-513	2 µM
BTN3del6	305-513	2.1 µM
BTN3 B30.2	310-513	No binding

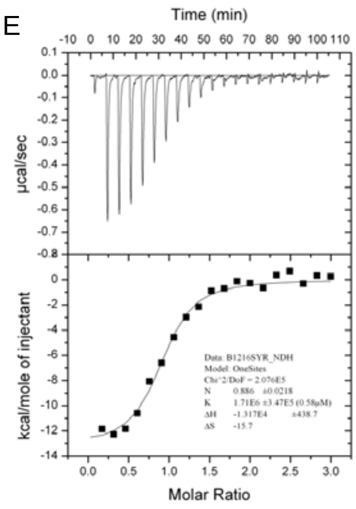

Figure S1 Interaction of BTN3A1 with periplakin

(A) Periplakin interacted with BTN3A1 in GST pull-down assays. Cos-7 cells were transfected with expression constructs PPL1-495 HA (top panel), PPL BamH1 (middle) and PPL B7 (bottom). Cell lysates were incubated with glutathione-Sepharose beads loaded with GST B30.2 domain fusion proteins. Eluted proteins were analyzed by IB with appropriate antibody together with input lysate representing 10% used in pull-downs. Bottom panel shows Coomassie stained gel of purified GST B30.2 fusion proteins

Lane 1. BTN2A1; 2. BTN3A3; 3. BTN3A1.

(B) Isothermal titration calorimetry was used to show interaction between periplakin and a series of deletion constructs covering the carboxyl-terminal juxta-membrane domain of BTN3A1, localising the interaction to amino acids 305-310.

(C) Periplakin interacts with BTN3A1v2 and with BTN1A1. An interaction of periplakin with the alternative splice variant 3A1v2, which lacks the B30.2 domain, was demonstrated by GST pull-down assay. GST pull-down assays with 1. BTN3A3, 2. BTN3A1, 3. BTN3A1v2 GST fusion proteins with periplakin deletion constructs. The site of interaction in the periplakin amino-terminal plakin domain was refined using a series of periplakin deletions to amino acids 396-495.

(D) Periplakin/ BTN3A1v2 interaction confirmed in transfected cells. IP of periplakin and BTN3A1v2 from 293T cell lysates. Cells were transfected with PPL1-495 HA and expression constructs for BTN1A1, BTN2A1 and BTN3A1v2 with carboxy terminal V5 epitope. Protein complexes were recovered by IP using anti-HA and analysed by IB using V5 antibody. BTN2A1 did not interact with periplakin, whereas BTN1A1 protein was detected in IP experiments.

(E) Isothermal titration calorimetry was used to confirm the interaction between BTN3A1v2 and PPL BamH1 proteins. An affinity of 0.58 μM was calculated for this interaction.

Supplementary Figure 2

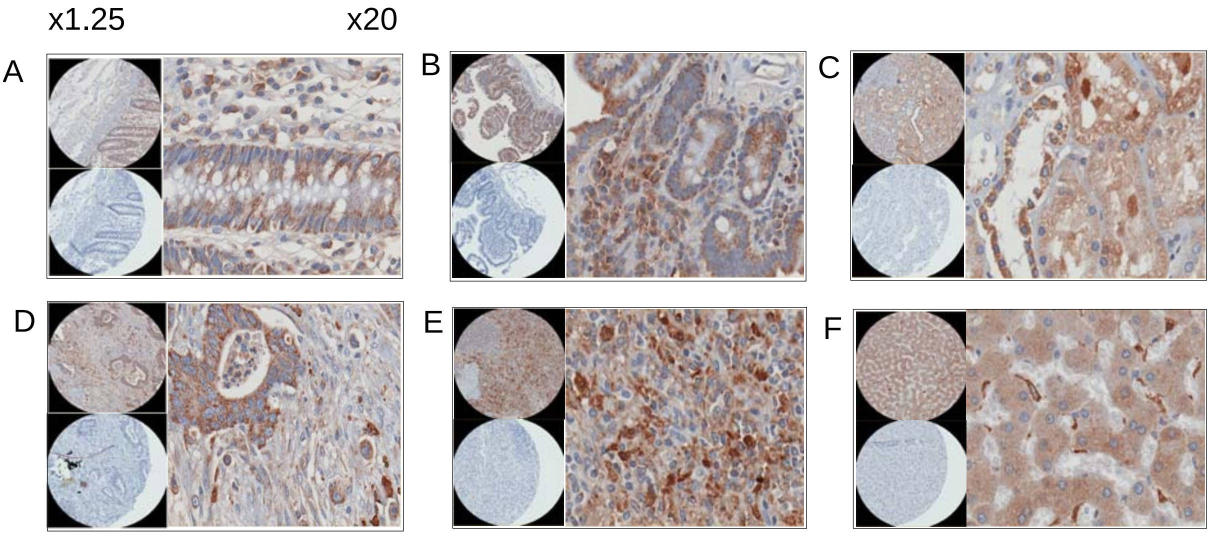

Figure S2

BTN3A staining of epithelium and macrophages detected by immunohistochemistry.

B6 antiserum stained cells in colon epithelium, small intestine and kidney, giving rise to a cytoplasmic and granular appearance (Fig.S2A, B and C respectively). Normal tissue staining was less intense than that observed in tumor cells, for example in colon (Fig.S2D) and breast tumor tissue (Fig.3E). Tissue macrophages in lung (Fig.3G), spleen and liver Kupffer cells stained strongly (FigS2 E and F respectively) as did endocrine tissue, including adrenal gland, pituitary and pancreas. Pancreatic staining showed more intense granular cytoplasmic staining localizing to Islets of Langerhans (Fig.3F). Some lymphoid and endothelial cell staining was detected. Serial tissue sections stained with rabbit polyclonal pre-immune serum were negative, as shown (lower inset). Results produced by Immunohistochemistry Group, Sanger Centre, Cambridge, UK.
